# Alterations of the Gut Microbiome and Fecal Metabolome in Colorectal Cancer: Implication of Intestinal Metabolism for Tumorigenesis

**DOI:** 10.3389/fphys.2022.854545

**Published:** 2022-05-04

**Authors:** Xinhao Du, Qing Li, Zhenzhen Tang, Li Yan, Ling Zhang, Qiao Zheng, Xianghao Zeng, Guimei Chen, Huawen Yue, Jun Li, Ming Zhao, Yuan-Ping Han, Xiangsheng Fu

**Affiliations:** ^1^ Department of Gastroenterology, The Affiliated Hospital of North Sichuan Medical College, Nanchong, China; ^2^ Department of Gastroenterology, Renmin Hospital of Wuhan University, Wuhan, China; ^3^ Department of Gastroenterology, Clinical Medical College and The First Affiliated Hospital of Chengdu Medical College, Chengdu, China; ^4^ The Center for Growth, Metabolism and Aging, College of Life Sciences, Sichuan University, Chengdu, China,

**Keywords:** colorectal cancer, fecal metabolome, gut microbiome, short-chain fatty acids, monosaccharides, biomarkers

## Abstract

**Objective:** The gut microbiota and its metabolites are important for host physiological homeostasis, while dysbiosis is related to diseases including the development of cancers such as colorectal cancer (CRC). In this study, we characterized the relationship of an altered gut microbiome with the fecal metabolome in CRC patients in comparison with volunteers having a normal colorectal mucous membrane (NC).

**Methods:** The richness and composition of the microbiota in fecal samples of 30 CRC patients and 36 NC controls were analyzed through 16S rRNA gene sequencing, and the metabolome was determined by ultra-performance liquid chromatography coupled to tandem mass spectrometry. Spearman correlation analysis was to determine the correlation between the gut microbiome and fecal metabolome in CRC patients.

**Results:** There were significant alterations in the gut microbiome and fecal metabolome in CRC patients compared with NC controls. Bacteroidetes, Firmicutes, Actinobacteriota, and Proteobacteria dominated the gut microbial communities at the phylum level in both groups. Compared with NC controls, CRC patients had a lower frequency of *Blautia* and *Lachnospiracaea* but a higher abundance of *Bacteroides fragilis* and *Prevotella*. Regarding the fecal metabolome, twenty-nine metabolites were identified as having significantly changed, showing increased levels of adrenic acid, decanoic acid, arachidonic acid, and tryptophan but a reduction in various monosaccharides in the fecal samples of CRC patients. Moreover, increased abundance of *Bacteroides fragilis* was strongly associated with decreased levels of monosaccharides, while *Blautia* was positively associated with the production of monosaccharides in the fecal samples.

**Conclusion:** These results highlight alterations of gut microbiota in association with certain metabolites in CRC progression, implying potential diagnostic and intervention potential for CRC.

## Introduction

Colorectal cancer (CRC), a serious malignant carcinoma, is the third leading cause of cancer-related deaths worldwide (Cao W et al., 2021). Increasing evidence has shown that the gut microbiota and its metabolites are closely associated with CRC carcinogenesis (Wu et al., 2021; Dalal et al., 2021; Duttaroy, 2021). Profiling of gut microbiota was explored for screening or predictive CRC biomarkers, and modulating the microbiota may have the potential for prevention or even treatment of CRC (Wong and Yu, 2019). The role of bacteria in CRC development has been supported by considerable studies. Compared with germ-free and conventional mice, it was found that the microbes transplanted from CRC patients could potentiate carcinogen-induced tumorigenesis, demonstrating the critical role of gut microbes in CRC development (Wong et al., 2017). An altered intestinal microbiome may serve as a “bacterial driver” to directly promote tumorigenesis in part through the induction of epithelial DNA damage and mutagenesis or by promoting so-called “passenger bacteria” to advantage the tumor microenvironment (Gagnière et al., 2016). On the other hand, bacterial biofilms may promote pro-carcinogenic activities for CRC initiation (Li S et al., 2017). Moreover, an altered gut microbiome and its metabolites may also elicit systemic and intestinal inflammation, a predominant reason for tumorigenesis at every stage (Cheng et al., 2020). Previous studies have demonstrated that specific microbes, such as *Fusobacterium nucleatum, Streptococcus bovis, Helicobacter pylori*, *Bacteroides fragilis*, and *Clostridium septicum*, contribute to CRC development. As a strong pathogen, *Fusobacterium nucleatum* could potentiate intestinal tumorigenesis through a TLR4/p-PAK1/b-catenin S675 cascade (Wu et al., 2018). Animal work has shown that *Streptococcus bovis* contributes to colorectal tumorigenesis by recruiting CD11b⁺TLR-4⁺ cells (Deng et al., 2020). Likewise, *enterotoxigenic Bacteroides fragilis* promoted CRC by inhibiting exosome-packaged miR-149-3pf (Cao Y et al., 2021), and *Helicobacter pylori* were associated with CRC, but the underlying mechanism remains unclear (Kim et al., 2017; Corredoira et al., 2017).

In addition to direct microbe-epithelial interactions, metabolites from the microbiome can profoundly impact host metabolism and pathogenesis. It has been reported that microbes may modulate cancer susceptibility or progression via metabolites (Peng et al., 2021). Short-chain fatty acids (SCFAs), the principal metabolites generated from gut microbial fermentation from insoluble dietary fiber or supplementation with SCFA-producing probiotics, can inhibit intestinal tumor development (Hou et al., 2022). Major SCFAs include acetate, butyrate, and propionate. Butyrate could inhibit the motility of colorectal cancer cells by deactivating Akt/ERK signaling (Li et al., 2017); acetate was shown to promote the expression of anti-inflammatory cytokines and reduce the generation of pro-inflammatory factors and NF-κB pathway activation in CRC cells (Tedelind et al., 2007); and propionate suppressed CRC growth by promoting the proteasomal degradation of euchromatic histone-lysine N-methyltransferase 2 (EHMT2) through HECT domain E3 ubiquitin protein ligase 2 (HECTD2) upregulation (Ryu et al., 2022). Secondary bile acids, lactate, trimethylamine N-oxide (TMAO), N-nitroso compounds, acetaldehyde, 4-hydroxyphenylacetic acid, phenylacetic acid, and phenol are related to CRC (Mohseni et al., 2020). Specifically, CpG-DNA produced by gut microbes can promote tumor cell proliferation and epithelial-mesenchymal transition and increase vasculogenic formation by targeting cellular receptors, leading to intestinal carcinogenesis (Song et al., 2021). Moreover, hydrogen sulfide was able to induce genomic DNA damage in host cells, which may lead to genomic instability in CRC (Attene-Ramos et al., 2007). Lactate is known to promote the proliferation, invasion, and migration of colon cancer cells (Chen et al., 2020). Thus, microbe-produced metabolites may either exert genotoxic and tumorigenic effects or protect the host through tumor-suppressive functions.

The combination of metagenomic sequencing analysis and metabolomics-based profiling of microbial metabolites can provide new insight into CRC biogenesis and prognosis. For instance, a recent review updated the notion that gut microbiome reprogramming in CRC patients is associated with alterations in the serum metabolome (Chen F et al., 2021). Another study using an untargeted approach found that polyamines made by gut microbes are associated with CRC (Yang et al., 2019). Of note, the same method was applied to find beneficial metabolites produced by intestinal symbionts (Dalal et al., 2021). Nevertheless, the particular association between microbes and metabolites in CRC development remains elusive. In the present study, we investigated fecal metabolome and microbiome profiles in CRC patients and compared them with healthy controls. Here, we identified a specific association between the gut microbiota and metabolite profiles, which may shed new light on potential applications for the prevention, diagnosis, and treatment of CRC.

## Materials and Methods

### Sample Collection

A cohort of 66 participants was enrolled from the Affiliated Hospital of North Sichuan Medical College (Nanchong, China) in this study, including 30 patients with sporadic CRC and 36 people with normal colorectal mucosa membrane (NC). The inclusion criteria were as follows: all participants over 18 years of age and received complete colonoscopy and who were newly diagnosed with colorectal cancer by pathological examination represented the CRC group, and healthy colorectal mucosa represented the NC group. All fecal samples were collected at the moment of diagnosis before any surgery or adjuvant treatment, immediately transported to the laboratory, and stored at -80 °C until further use. Exclusion criteria were set including immunodeficiency, cardiopathy, diabetes, hypertension, other malignant tumors or other severe gastroenterological diseases, antibiotic treatment in the last 2 months, and regular treatment with nonsteroidal anti-inflammatory drugs, probiotics, or statins. Individuals who received preoperative chemotherapy or radiation therapy were also excluded from the study. Clinical data, such as age, sex, tumor stage, tumor location, and tumor differentiation, were acquired according to hospital records. Prior to enrollment, informed consent was obtained from all participants. The study was approved by the Institutional Review Board of the Affiliated Hospital of North Sichuan Medical College (Nanchong, China).

### Fecal DNA Extraction for Microbiome Analysis

DNA from fecal samples was isolated utilizing the E. Z.N.A.® Soil DNA kit (Omega Bio-Tek, Norcross, GA, U.S.), followed by the quantification of DNA concentration and purity measurement with a NanoDrop2000 UV–vis spectrophotometer (Thermo Scientific, Wilmington, United States).

### High-Throughput 16S Ribosomal RNA Gene Sequencing

The hypervariable region V4 of the bacterial 16S rRNA gene was amplified with the forward primer 515F (5′-GTGCCAGCMGCCGCGG-3′) and reverse primer 806R (5′-GGACTACHVGGGTWTCTAAT-3′) by an ABI GeneAmp®9700 PCR thermocycler (ABI, CA, United States). The amplicons were purified and pooled in equimolar amounts and paired-end sequenced on an Illumina MiSeq PE300 platform/NovaSeq PE250 platform (Illumina, San Diego, United States) according to the standard protocols by Majorbio Bio-Pharm Technology Co., Ltd. (Shanghai, China).

### Processing of Sequencing Data

The raw 16S rRNA gene sequencing reads were demultiplexed, quality-filtered by fastp version 0.20.0 (Chen et al., 2018), and merged by FLASH version 1.2.7 (Magoč and Salzberg, 2011) with the following criteria: 1) the 300 bp reads were truncated at any site receiving an average quality score of <20 over a 50 bp sliding window, and the truncated reads shorter than 50 bp or the reads containing ambiguous characters were discarded; 2) only overlapping sequences longer than 10 bp were assembled according to their overlapped sequence. The maximum mismatch ratio of the overlap region is 0.2. Reads that could not be assembled were discarded; and 3) samples were distinguished according to the barcode and primers, and the sequence direction was adjusted, with exact barcode matching and two nucleotides mismatched in primer matching.

Operational taxonomic units (OTUs) with a 97% similarity cutoff were clustered using UPARSE version 7.1 (Edgar, 2013), and chimeric sequences were identified and removed. To minimize the variation in sequencing depth between samples, an average and rounded rarefied OTU value table was constructed by calculating the average of 100 average resampled OTU subsets (minimum sequencing depths of <90%) for further study. The taxonomy of each OTU representative sequence was analyzed by RDP Classifier version 2.2 (Wang et al., 2007) against the 16S rRNA database (e.g., Silva v138) using a confidence threshold of 0.7.

### Diversity Indices

The Shannon index and Simpson index were calculated to characterize the α-diversity. The Shannon index is derived using the following equation:

$$H=-\overset{S_{obs}}{\underset{i=1}{\sum }}\frac{n_{i}}{N}ln\frac{n_{i}}{N},$$

whereH = Shannon index.S_obs_ = number of OTUs. $n_{i}$ = number of sequences contained in the i ^th^ OTU. ln = natural log.N = all numbers of sequences.

The higher the Shannon index, the higher the microbiota diversity.

The Simpson index was calculated using the following formula:

$$D=\frac{\sum_{i=1}^{S_{obs}}n_{i}\left(n_{i}-1\right)}{N\left(N-1\right)},$$

whereD = Simpson index.S_obs_ = number of OTUs. $n_{i}$ = number of sequences contained in the i ^th^ OTU.N = all numbers of sequences.

As biodiversity increases, the Simpson index decreases.

#### Sample Preparation for Metabolomics Analysis

Fecal samples were thawed on ice. Approximately 5 mg of each lyophilized sample was weighed and transferred to a new 1.5 ml tube. Then, 25 μL of water was added, the sample was homogenized with zirconium oxide beads for 3 min, and 120 μL of methanol containing internal standard was extracted for gut metabolites. The sample was homogenized for another 3 min and then centrifuged at 18,000 rpm for 20 min. Then, 20 μL of supernatant was transferred to a 96-well plate. The following procedures were performed on an Eppendorf epMotion Workstation (Eppendorf Inc., Hamburg, Germany). A total of 20 μL of freshly prepared derivative reagents was added to each well. The plate was sealed, and the derivatization was carried out at 30°C for 60 min. After derivatization, 330 μL of ice-cold 50% methanol solution was added to dilute the sample. Then the plate was stored at -20°C for 20 min, followed by centrifugation at 4,000 g at 4°C for 30 min. A total of 135 μL of supernatant was transferred to a new 96-well plate with 10 μL of internal standards in each well. Serial dilutions of derivatized stock standards were added to the left wells. Finally, the plates were sealed for analysis.

### Ultra-performance Liquid Chromatography Coupled to Tandem Mass Spectrometry (UPLC–MS/MS) Analysis

A 5 μL aliquot of the pretreated sample was injected into a 100 mm × 2.1 mm, 1.7 μM BEH C18 column (Waters, Milford, United States) on a UPLC–MS/MS station (Waters, Milford, United States). The column temperature was set at 40°C, and the flow rate was 0.40 ml/min. The mobile phase consisted of two solutions, A (water with 0.1% formic acid) and B (acetonitrile), and was eluted in the following proportions: the mode involved 5% acetonitrile for 0-1 min, 5–78% for 1–11 min, 78–95% for 11–13.5 min, and 95–100% for 13.5–14 min; the concentration was then held at 98% for 2 min, returned to 5% for 16–16.1 min, and finally held at 5% for 16.1–18 min. All analyses were acquired with a locking spray to ensure accuracy and reproducibility.

### Quality Control and Data Processing

To achieve reliable data, quality control (QC) samples are routinely used in our metabolomics platform. In addition to the quality controls, conditioning samples and solvent blank samples were also required for obtaining optimal instrument performance. QC samples were used to assess the reproducibility and reliability of the UPLC–MS system. The QC sample, made by mixing and blending equal volumes (10 μL) of each fecal sample, was used to estimate a mean profile representing all the analytes encountered during analysis. Reagent blank samples are a mixture of solvents used for sample preparation and are commonly processed using the same procedures as the samples to be analyzed. The calibrators consist of a blank sample (matrix sample processed without internal standard), a zero sample (matrix sample processed with internal standard), and a series of seven concentrations covering the expected range for the metabolites present in the specific biological samples. To diminish analytical bias within the entire analytical process, the samples were analyzed in group pairs, but the groups were analyzed randomly. The QC samples, calibrators, and blank samples were analyzed across the entire sample set.

The raw data files generated by UPLC–MS/MS were processed using MassLynx software (v4.1, Waters, Milford, MA, United States) to perform peak integration, calibration, and quantitation for each metabolite. The self-developed platform iMAP (v1.0, Metabo-Profile, Shanghai, China) was used for statistical analyses, including PCA, OPLS-DA, univariate analysis, and pathway analysis.

### Quantitation of Metabolites

Mass spectrometry-based quantitative metabolomics refers to the determination of the concentration of a substance in an unknown sample by comparing the unknown to a set of standard samples of known concentration (i.e., calibration curve). The calibration curve is a plot of how the analytical signal changes with the concentration of the analyte (the substance to be measured). For most analyses, a plot of instrument response vs. concentration will show a linear relationship. This yields a model described by the equation y = ax + b, where y is the instrument response, e.g., peak height or area, a represents the slope/sensitivity, and b is a constant that describes the background. The analyte concentration (x) of unknown samples may be calculated from this equation.

### Statistical Analysis

The differences in alpha diversity between the CRC and NC groups were analyzed by using Student’s t tests. We used ANOSIM to compare differences in beta diversity between groups, using weighted UniFrac for categorical variables and the Mann–Whitney-Wilcoxon test to compare the abundance of microbiota between the CRC and NC groups. A metagenomic biomarker discovery approach was employed with LEfSe (linear discriminant analysis coupled with effect size measurement), which performed a nonparametric Wilcoxon sum-rank test followed by LDA analysis using online software (http//huttenhower.sph.harvard.edu/galaxy/) to assess the effect size of each differentially abundant taxon.

Orthogonal projection to latent structure discriminant analysis (OPLS-DA) was performed to examine the overall fecal metabolite distribution in each group. The qualities of all OPLS-DA models were assessed with R^2^X (the total variation explained by the model) and Q^2^ (denoting the predictability of the model). The significance of the models was further validated by univariate statistical analyses including Student’s t test, Mann–Whitney-Wilcoxon test, ANOVA, and correlation analysis. Moreover, we also used the intersection of univariate analysis and OPLS-DA models to identify the metabolic biomarkers of CRC.

The *p*-values were adjusted for multiple comparisons using the Benjamini–Hochberg false discovery rate (FDR). Both raw *p*-values (*P*) and *P*-adjusted FDR *Q*-values (*Q*) are reported. Microbes with a raw *p*-value of less than 0.05 were considered significant since FDR correction can increase the risk of false-negative conclusions. To report the magnitude of the difference between the groups as well as to consider the relatively small sample size, effect size and 95% confidence intervals were also calculated. The meaningful metabolites were listed according to the following parameters: a univariate *p*-value of <0.05, a *Q*-value of <0.25, and a multivariate variable importance in the projection (VIP) value of >1.0. We used receiver-operating characteristic (ROC) curves to evaluate the predictive ability of differential metabolites for CRC diagnosis. To avoid overfitting the data with logistic regression models, we used leave-one-out cross-validation. For correlation analysis, we conducted Spearman’s rank test as implemented in the R package (v 3.2.0). All graphs were generated using the Adobe Illustrator CC 2017 release or GraphPad Prism 7.

## Results

### Pathological Characteristics of the Human Subjects

All patients and volunteers were Han Chinese from Sichuan Province, China, with comparable eating habits. The clinicopathological characteristics are listed in Table 1. Both groups were age- and sex-matched, and they had similar body mass indexes (BMIs). Furthermore, the two groups had similar lifestyle factors, and most subjects did not smoke and occasionally drink.

**TABLE 1:** The basic characteristics of patients.

	NC (*n* = 33)	CRC (*n* = 30)	*p*-value
Gender			
Female (n)	20	18	0.204
Male (n)	13	12	
Average age (year)	59.27	56.67	0.222
Location			
Right location	NA	5	
Left location	NA	25	
TNM staging			
I	NA	2	
II		12	
III		14	
IV		2	
Differentiation			
WD	NA	6	
MPD		24	
Metastasis			
Non-LNM	NA	13	
LNM		17	
Smoking			
Absence	25	24	0.6916
Presence	8	6	
Drinking			
Never	10	8	0.5907
Occasionally	22	20	
Frequently	1	2	

AD, adenoma; CRC, colorectal cancer; LNM, lymph node metastasis; MPD, moderately and poorly differentiated; NA, not applicable; NC, human with normal colorectal mucous membrane; Non-LNM, non-lymph node metastasis; WD, well-differentiated.

### Decreased Diversity of the Fecal Microbiome in CRC Patients

**FIGURE 1:**
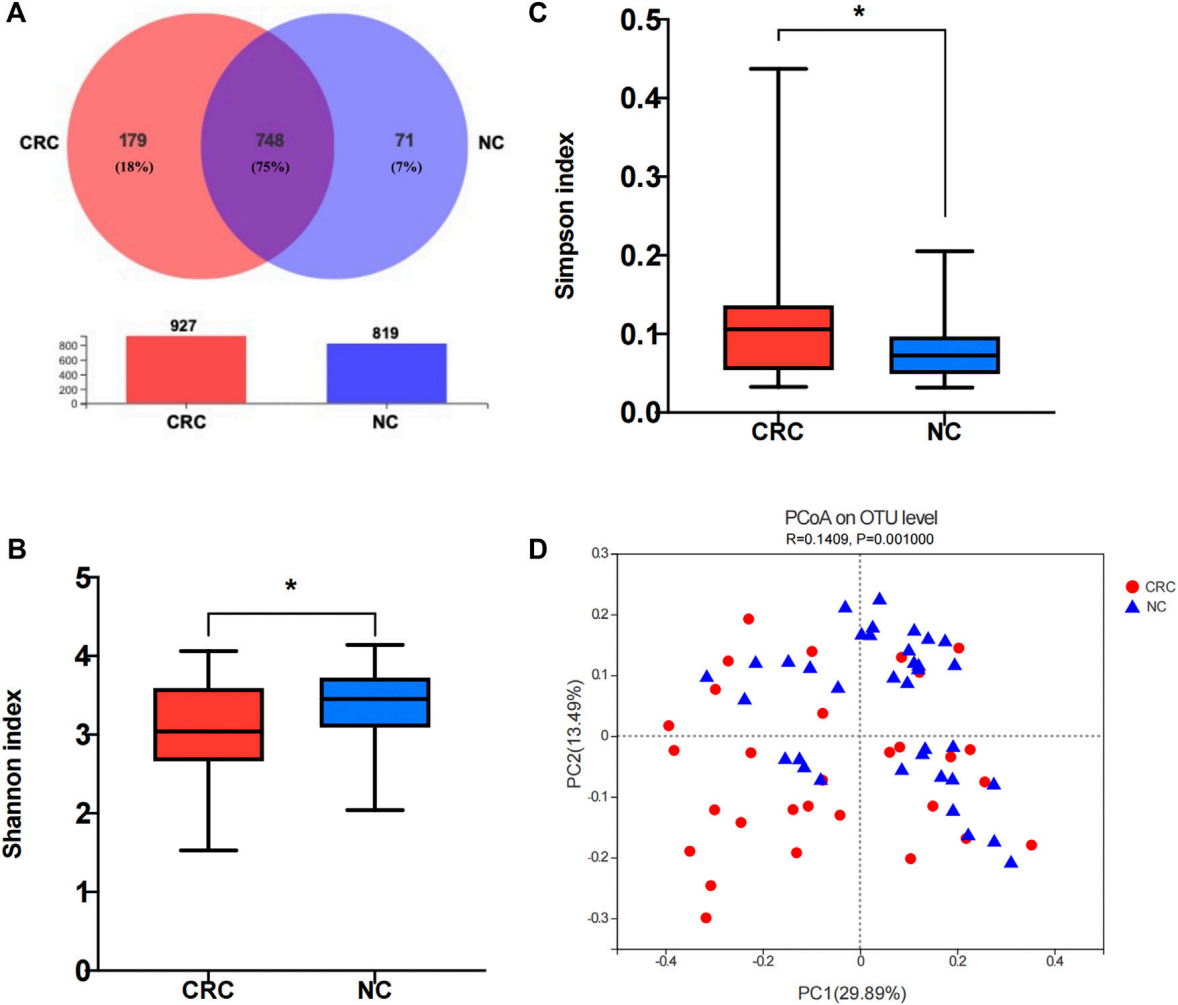
Comparison of the fecal microbiome composition between the CRC and NC groups. **(A)** Venn diagram of operational taxonomic units (OTUs) in CRC patients and NC controls. **(B,C)** α-diversity of 16S rRNA genes in the gut microbiota of CRC patients and NC controls. **(D)** Principal coordinate analysis (PCoA) of a weighted UniFrac distance analysis. NC, normal colorectal mucous membrane; CRC, colorectal cancer.

To measure the diversity of the fecal microbiota of CRC patients and healthy volunteers, we performed 16S rDNA sequencing and obtained a total of 3,815,908 high-quality sequences with an average length of 255 for each sample **(** Supplementary Table S1 **)**. A total of 998 OTUs were identified, and the coverage values approached 99.99% in both groups, indicating that the sequencing depth was sufficient for the discovery and investigation of the microbiota. The sequences were then clustered into operational taxonomic units (OTUs), with 74.9% (748 in 998) shared by the two groups and 179 unique to the CRC group (Figure 1A). Further analysis showed a significant difference in the Shannon index (*p* = 0.04) (Figure 1B) and Simpson index (*p* = 0.04) (Figure 1C) between the CRC and NC groups, demonstrating a remarkably lower α-diversity in CRC patients than that in NC controls (Supplementary Table S2). *ß*-diversity refers to species diversity among different environmental groups. A significant difference in *ß*-diversity was obtained using the weighted UniFrac distance statistic (qualitative, ADONIS *p* = 0.001) (Figure 1D). The observed differences in α- and *ß*-diversity indicated the imbalanced structure of the intestinal flora in CRC patients compared with NC controls.

### Alterations in the Taxa Between Fecal Specimens of CRC Patients and NC Controls

**FIGURE 2:**
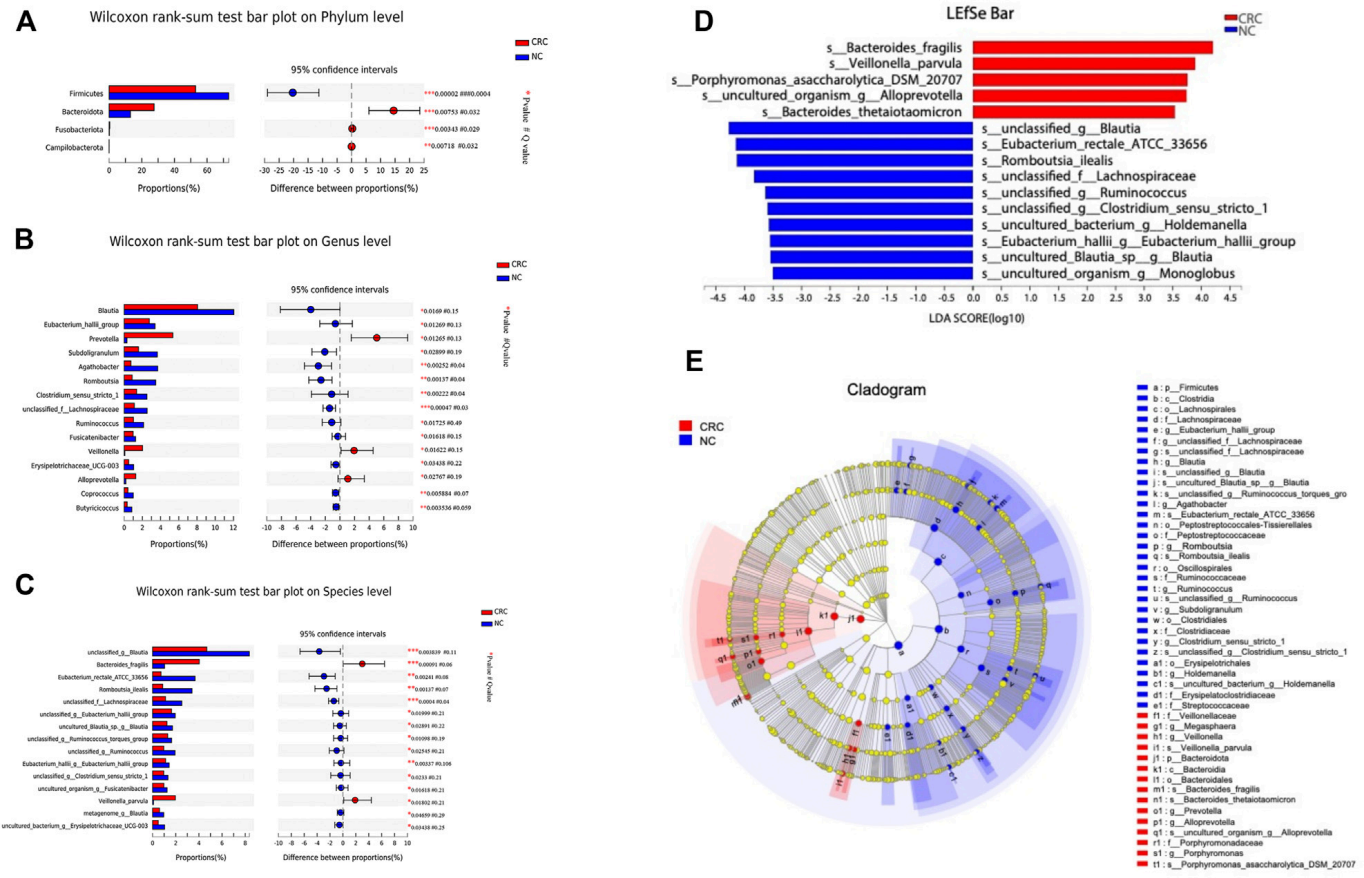
Comparison of the abundance of fecal microbiota by CRC and NC groups. **(A-C)** Relative abundance of the bacterial composition in the CRC and NC groups at the phylum, genus, and species levels. **(D)** LEfSe analysis of enriched bacterial taxa in the gut microbiota of the CRC and NC groups (LDA>3.5 of LEfSe). **(E)** Cladogram representation of the taxonomic differences between the CRC and NC groups.

To identify specific microbiota species or genera associated with CRC and compare the composition of fecal microflora between CRC and NC groups, Welch’s *t* test was performed at different taxonomic levels. At the phylum level, Bacteroidetes, Firmicutes, Actinobacteriota, and Proteobacteria dominated the gut microbial communities in both groups. Compared with NC controls, CRC patients had fewer Firmicutes (*p* = 0.00002) but higher levels of Bacteroidota (*p* = 0.007), Fusobacteriota (*p* = 0.003), and *Campylobacter* (*p* = 0.007). Each differential phylum remained significant after FDR correction (Figure 2A). At the genus level, we found that *Prevotella* was increased (*p* = 0.01) in the CRC group, which most studies showed to be CRC-associated pathobionts (Liu W et al., 2021), while *Blautia*, *Eubacterium hallii group*, *Subdoligranulum*, *Agathobacter*, *Romboutsia,* and *Clostridium sensus tricto 1* were significantly decreased (*p* < 0.05). After FDR correction, only *Agathobacter*, *Romboutsia,* and *Clostridium sensus tricto 1* reached a significant difference (Figure 2B). Prior to FDR correction, some bacteria showed *p*-values lower than 0.05 at the species level. The abundance of *Bacteroides fragilis* (*p* = 0.0009) and *Veillonella parvula* (*p* = 0.01) was markedly enriched in CRC patients. Notably, *Veillonella parvula* is a common and abundant member of the oral microbiome and possesses important metabolic pathways that utilize lactate as an energy source (Mashima et al., 2021), but there have been no previous reports that this bacterium was enriched in CRC patients. Some bacteria with probiotic properties, such as *Blautia*, *Eubacterium rectale ATCC33656,* Lachnospiraceae*,* and *Eubacterium hallii*, were significantly enriched in NC controls (Figure 2C).

To determine the role of fecal microflora in CRC progression, we detected the fecal microbial communities in various differentiation and pathological tumor-node-metastasis (pTNM) stages. However, there was no significantly different microbiota in fecal samples among CRC patients at TNM stage I, stage II, stage III, and stage IV (*p* > 0.05, Supplementary Figure S1A). Likewise, we did not find statistically significantly different microbiota between the well-differentiated and moderately/poorly differentiated CRC fecal specimens (*p* > 0.05, Supplementary Figure S1B).

Next, we performed a linear discriminant analysis effect size (LEfSe) analysis to identify specific taxa with the varied abundance that may make them potential diagnostic biomarkers. In total, 14 species were identified with LDA scores >3.5 (Figure 2D). According to LEfSe analysis, the CRC group was enriched with five species, including *Bacteroides fragilis*, *Veillonella parvula*, *Porphyromonas asaccharolytica DSM 20707*, *uncultured organism Alloprevotella,* and *Bacteroides thetaiotaomicron*, while the NC group was enriched with nine species, including *unclassified Blautia*, *Eubacterium_rectale ATCC 33656*, *unclassified* Lachnospiraceae*,* and *unclassified Ruminococcus*. A cladogram from phyla to species is shown in Figure 2E. These results showed reduced probiotic species and an increased quantity of facultative pathogenic bacteria in patients with CRC. Taken together, these data indicate changes in the gut microbiota in CRC patients. Specifically, CRC is associated with a reduction in the biodiversity of gut bacteria compared with the NC controls. Thus, CRC-specific fecal microbial communities may have pathological relevance as potential diagnostic markers.

### Metabolomic Signature of Fecal Samples From CRC Patients and NC Controls

**FIGURE 3:**
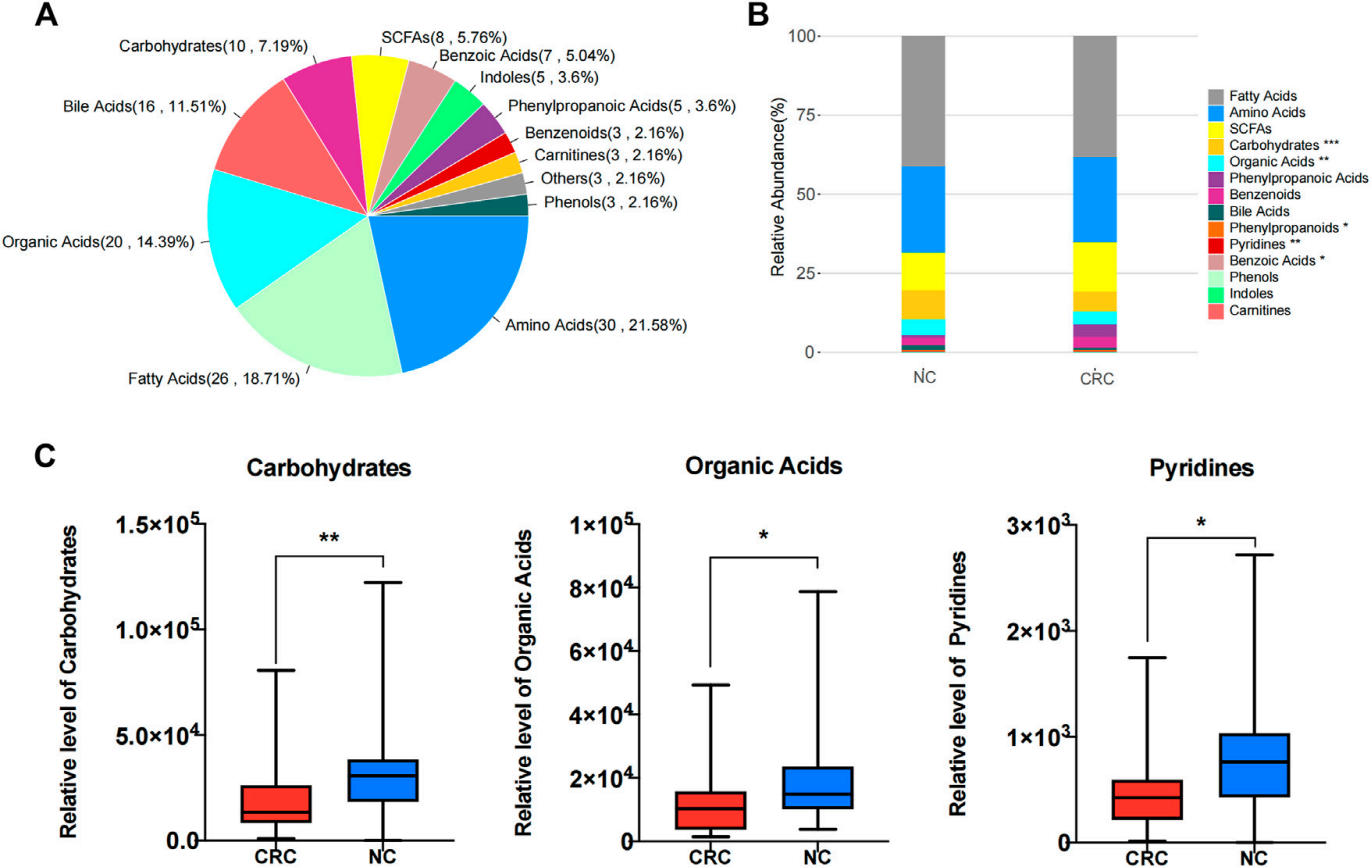
Metabolomic signature of fecal samples from CRC and NC groups. **(A)** Pie chart illustrating the abundance ratio of different classes of metabolites detected by targeted metabolic profiling in fecal samples from CRC patients and NC controls. **(B)** Stacked bar plot showing the relative abundance of different classes of metabolites in both the control CRC and NC groups. *p < 0.05, **p < 0.01, ***p < 0.001. **(C)** Aggregate values for metabolite categories from CRC patients and NC controls. *p < 0.05, **p < 0.01.

It was described that the metabolites produced by gut microbiota could modulate host immune systems (Ejtahed et al., 2020; Parker et al., 2020). To identify the metabolomic signature of CRC patients, we compared the fecal metabolomics profiles of CRC patients and NC controls through in-depth targeted metabolomics analysis of fecal contents by UPLC–MS/MS. In total, 139 metabolites were identified, followed by classification into 12 categories (Figure 3A). Specifically, we identified short-chain fatty acids (SCFAs), benzoic acids, indole, phenylpropanoic acid, benzenoid, carmitines, phenol, amino acids, fatty acids, organic acids, bile acids, and carbohydrates from the fecal samples. Compared with NC controls, three categories, including carbohydrate (*p* = 0.001173), organic acids (*p* = 0.016787), and pyridines (*p* = 0.014594), were significantly decreased (Figures 3B, C; Supplementary Figure S2), which is in line with the decreased bacterial diversity. Of interest is that many gut metabolites are actually acidic components derived from bacterial fermentation. The inherent trend within the metabolic data of the CRC and NC groups was distinguished through a principal component analysis (PCA) algorithm. The score plot of the PCA showed that the CRC group had a clear tendency of separation from the NC group (Supplementary Figure S3A). To better discriminate the fecal metabolic profiles of the NC group and the CRC group, a supervised orthogonal partial least squares discriminant analysis (OPLS-DA) was constructed, which can enhance the separation between groups of observations and improve the interpretation of models. To this end, a clear separation with some overlap was observed between these two groups in the selected OPLS-DA model (Supplementary Figure S3B). The goodness of fit (R^2^Y) and prediction ability (Q^2^) of this model were 0.647 and 0.258, respectively, suggesting good fitness and predictive ability of the OPLS-DA model (Supplementary Figure S3C).

### Identification of Metabolic Biomarkers

**FIGURE 4:**
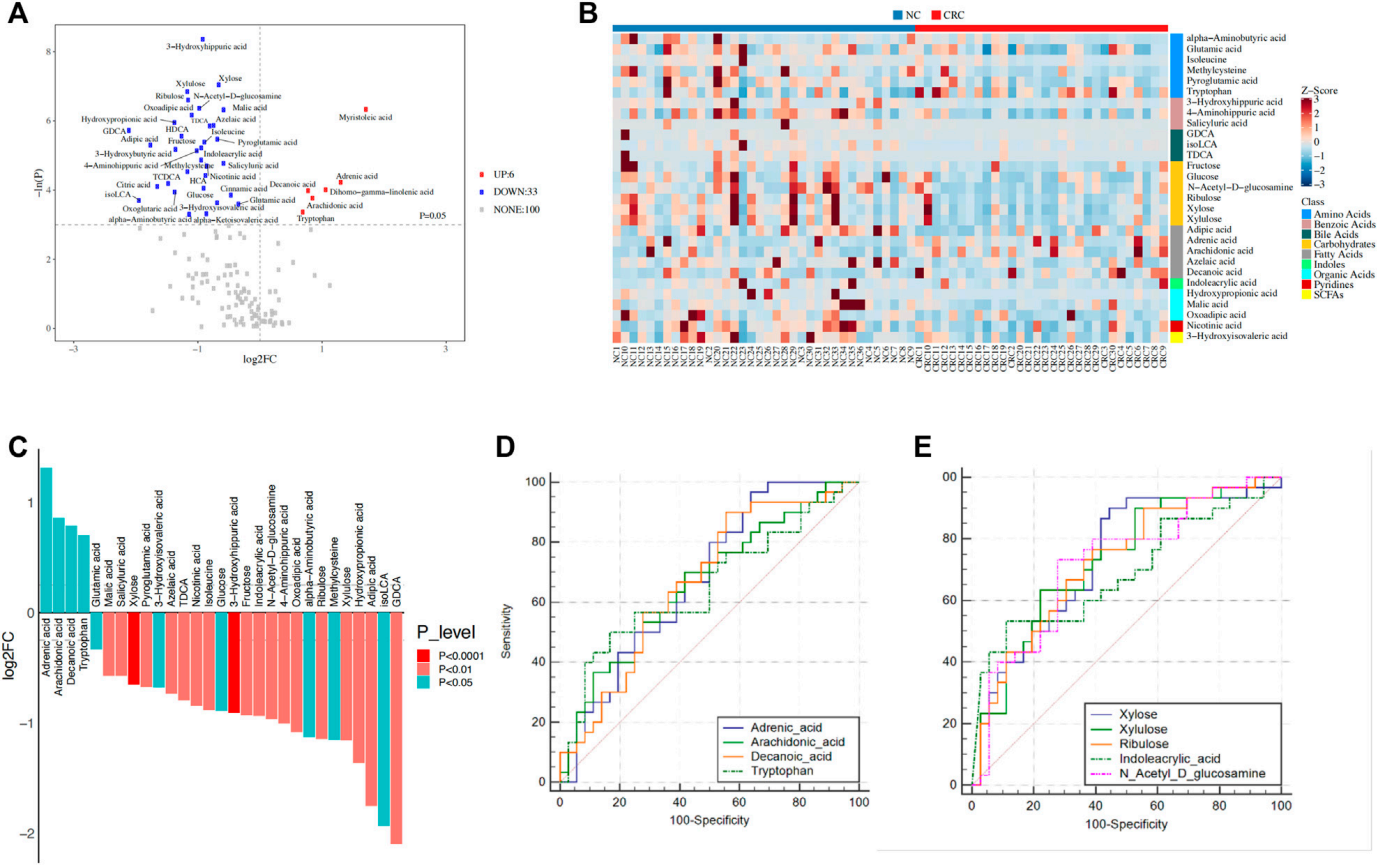
Identification of metabolites related to CRC. **(A)** Identification of dysregulated metabolites in CRC patients and NC controls. *p*-values were calculated using the Mann–Whitney-Wilcoxon test. **(B)** Heatmap showing the relative content of dysregulated metabolites. **(C)** The 29 dysregulated metabolites were selected based on the intersection of univariate analysis and OPLS-DA models. Significance was determined by the Mann–Whitney-Wilcoxon test. **(D,E)** ROC curve analysis of upregulated metabolites **(D)** and the top five downregulated metabolites **(E)** in the CRC group. OPLS-DA, orthogonal partial least squares discriminant analysis; ROC, receiver operating characteristic; A, colorectal cancer group; B, normal colorectal mucous membrane group.

Based on the comparison of metabolites between the CRC and NC groups, 53 individual metabolites that differed in abundance were identified (Supplementary Figure S4). To avoid intergroup biases and an uneven sample distribution, univariate analysis with the Mann–Whitney-Wilcoxon test (*p* ≤ 0.05 and |log2FC| ≥ 0) further narrowed down the metabolites to 39 identities, including 33 downregulated and six upregulated metabolites (Figure 4A).

To further identify the metabolic biomarkers of CRC, taking the intersection of univariate analysis and orthogonal partial least squares discriminant analysis (OPLS-DA) models, a total of 29 metabolites were selected as potential biomarkers (25 metabolites were downregulated, and four metabolites were upregulated in the CRC group) (Table 2; Figure 4B). First, five long-chain fatty acids were distinguished, and three fatty acids, adrenic acid (*p* = 0.01, *Q* = 0.08), arachidonic acid (*p* = 0.02, *Q* = 0.09), and decanoic acid (*p* = 0.01, *Q* = 0.08), were upregulated in the CRC group (Figure 4C). In particular, the data are in agreement with a previous report showing that decanoic acid may serve as a biomarker for the diagnosis of early CRC (Uchiyama et al., 2017). SCFAs, such as acetate, propionate, and butyrate, are beneficial and downregulated in colorectal cancer (Hou et al., 2022), while our results found that 3-hydroxyisovaleric acid (*p* = 0.02, *Q* = 0.10), which is a natural catabolite of leucine and can regulate excessive inflammation (Cavallucci and Pani, 2021), was actually downregulated in the CRC group. It is generally known that bacterial enzymes convert tryptophan into indole and its derivatives (Kumar et al., 2021), and our results showed a significantly lower content of indoleacrylic acid (*p* = 0.007, *Q* = 0.053) and a higher content of tryptophan (*p* = 0.03, *Q* = 0.13) in the CRC group. This paradoxical phenomenon might reflect gut dysbiosis that impaired the tryptophan metabolic pathway. In agreement with increased fatty acids in the feces of CRC, short-chain carbohydrates, i.e., monosaccharides such as xylulose (*p* = 0.001, *Q* = 0.035), xylose (*p* = 0.0009, *Q* = 0.035), ribulose (*p* = 0.01, *Q* = 0.08), fructose (*p* = 0.005, *Q* = 0.04), and glucose (*p* = 0.01, *Q* = 0.08) were decreased, which may be due to increased fermentation by gut microbes in CRC. Moreover, the top three identified metabolomic pathways in CRC (*p* < 0.05) were starch and sucrose metabolism, pentose and glucuronate interconversions, and aminoacyl-tRNA biosynthesis, of which the starch and sucrose metabolism pathways were the most notable (Supplementary Table S3; Supplementary Figure S5).

**TABLE 2:** The potential metabolic biomarkers in CRC and NC groups.

Metabolite	Class	HMDB	KEGG	Uni_P	Uni_FDR	FC	log2FC	OPLSDA_VIP	Negatively associated bacteria	Positively associated bacteria
3-Hydroxyisovaleric acid	SCFAs	HMDB0000754	NA	0.0265	0.1053	0.6258	−0.6763	1.7164	Campilobacterota	NA
									Euryarchaeota	
4-Aminohippuric acid	Benzoic acids	HMDB0001867	D06890	0.0059	0.0431	0.4988	−1.0036	2.0604	Campilobacterota	Firmicutes
									Euryarchaeota	
									Bacteroidota	
Adipic acid	Fatty acids	HMDB0000448	C06104	0.005	0.0431	0.2969	−1.7521	1.9343	NA	Desulfobacterota
Adrenic acid	Fatty acids	HMDB0002226	C16527	0.0147	0.081	2.4847	1.313	1.1435	Desulfobacterota	Patescibacteria
										Chloroflexi
Arachidonic acid	Fatty acids	HMDB0001043	C00219	0.0231	0.0973	1.8146	0.8596	1.8683	Desulfobacterota	Patescibacteria
Azelaic acid	Fatty acids	HMDB0000784	C08261	0.0028	0.0363	0.6015	−0.7333	1.3316	Campilobacterota	Firmicutes
									Bacteroidota	
Fructose	Carbohydrates	HMDB0000660	C02336	0.0054	0.0431	0.5241	−0.9321	1.2895	Bacteroidota	Firmicutes
									Desulfobacterota	Patescibacteria
Glucose	Carbohydrates	HMDB0000122	C00221	0.0174	0.0864	0.5383	−0.8935	1.8352	Verrucomicrobiota	Firmicutes Patescibacteria
									Euryarchaeota	
									Synergistota	
									Bacteroidota	
									Desulfobacterota	
Ribulose	Carbohydrates	HMDB0000621	C00309	0.0014	0.0358	0.4524	−1.1445	2.0969	Euryarchaeota	Firmicutes Patescibacteria
									Synergistota	
									Bacteroidota	
									Campilobacterota	
Xylose	Carbohydrates	HMDB0000098	C00181	9.0E-4	0.0358	0.6365	−0.6519	2.1046	Verrucomicrobiota	Firmicutes Patescibacteria
									Euryarchaeota	
									Synergistota	
									Bacteroidota	
									Desulfobacterota	
									Campilobacterota	
Xylulose	Carbohydrates	HMDB0001644	C00310	0.0011	0.0358	0.4484	−1.1571	2.1097	Euryarchaeota	Firmicutes Patescibacteria
									Synergistota	
									Bacteroidota	
									Campilobacterota	
									Methylomirabilota	
Decanoic acid	Fatty acids	HMDB0000511	C01571	0.0187	0.0867	1.7265	0.7879	1.2096	NA	Synergistota
										Actinobacteriota
Glycodeoxycholic acid	Bile acids	HMDB0000631	C05464	0.0033	0.0379	0.2335	−2.0986	1.0268	Proteobacteria	Firmicutes
Hydroxypropionic acid	Organic acids	HMDB0000700	C01013	0.0026	0.0363	0.3883	−1.3649	1.392	Bacteroidota	Firmicutes
									Desulfobacterota	Patescibacteria
Indoleacrylic acid	Indoles	HMDB0000734	NA	0.0077	0.0534	0.5234	-0.9341	1.0677	Fusobacteriota	NA
									Campilobacterota	
isoLCA	Bile acids	HMDB0000717	C17658	0.0248	0.1012	0.2611	−1.9372	1.2815	Desulfobacterota	Proteobacteria
										Patescibacteria
alpha-Aminobutyric acid	Amino acids	HMDB0000452	C02356	0.0369	0.1317	0.4579	−1.127	1.042	Verrucomicrobiota	Firmicutes Patescibacteria
									Euryarchaeota	
									Bacteroidota	
									Desulfobacterota	
Glutamic acid	Amino acids	HMDB0000148	C00025	0.0274	0.1059	0.7932	-0.3343	1.7331	Campilobacterota	Firmicutes
									Euryarchaeota	
									Bacteroidota	
Isoleucine	Amino acids	HMDB0000172	C00407	0.0046	0.0425	0.542	−0.8835	1.0266	Bacteroidota	Firmicutes
									Desulfobacterota	Patescibacteria
										Actinobacteriota
Malic acid	Organic acids	HMDB0000156	C00149	0.0018	0.0358	0.6719	−0.5736	1.502	Campilobacterota	NA
Tryptophan	Amino acids	HMDB0000929	C00078	0.0346	0.1301	1.6275	0.7026	2.07	Bacteroidota	Patescibacteria
									Desulfobacterota	Methylomirabilota
3-Hydroxyhippuric acid	Benzoic acids	HMDB0006116	NA	2.0E-4	0.0326	0.5327	−0.9087	1.5298	Bacteroidota	Firmicutes
										Patescibacteria
Methylcysteine	Amino acids	HMDB0002108	NA	0.0108	0.0651	0.4489	−1.1556	1.7201	Euryarchaeota	Firmicutes
									Bacteroidota	Patescibacteria
									Desulfobacterota	
N-Acetyl-D-glucosamine	Carbohydrates	HMDB0000215	C00140	0.0017	0.0358	0.5123	−0.965	1.3566	Synergistota	Firmicutes
									Bacteroidota	Patescibacteria
									Campilobacterota	
Nicotinic acid	Pyridines	HMDB0001488	C00253	0.0092	0.058	0.5566	−0.8452	1.7317	Euryarchaeota	NA
Oxoadipic acid	Organic acids	HMDB0000225	C00322	0.0021	0.0363	0.4711	−1.0859	1.2364	Bacteroidota	Firmicutes
										Patescibacteria
Pyroglutamic acid	Amino acids	HMDB0000267	C01879	0.0042	0.0418	0.6268	−0.6739	1.2421	Campilobacterota	Firmicutes
									Bacteroidota	
Salicyluric acid	Benzoic acids	HMDB0000840	C07588	0.0085	0.0562	0.6716	−0.5744	1.2091	NA	NA
Tauroursodeoxycholic acid	Bile acids	HMDB0000896	C05463	0.0029	0.0363	0.5758	−0.7964	1.1127	Verrucomicrobiota	Firmicutes
									Synergistota	Patescibacteria
									Bacteroidota	

FC, fold change, is calculated as CRC group/NC group.

To summarize the metabolism alterations according to the progressive stage of CRC, we classified the cancer stage according to the TNM staging system of the eighth edition of the American Joint Committee on Cancer (AJCC) cancer staging manual and further identified the differential metabolites in different TNM stages. Previously, we found that monosaccharides were significantly reduced in the CRC group, but this phenomenon was not found in the progressive stage of CRC. We found that the SCFAs (butyric acid, isovaleric acid, ethylmethylacetic acid, and acetic acid), carnitine, indole-3-carboxylic acid, 4-hydroxyphenylpyruvic acid, and penylpyruvic acid decreased gradually with tumor progression (Supplementary Table S4). However, the specific mechanism underlying these differences requires further study.

Next, we assessed the potential of the dysregulated metabolites to serve as CRC diagnostic biomarkers. A ROC curve analysis indicated that the four upregulated metabolites, including adrenic acid (area under the curve (AUC), 0.676; *p* = 0.01), arachidonic acid (AUC, 0.663; *p* = 0.02), decanoic acid (AUC, 0.669; *p* = 0.002), and tryptophan (AUC, 0.652; *p* = 0.03), were significantly associated with CRC patients (Figure 4D) while the five downregulated metabolites, including xylose (AUC, 0.735; *p* = 0.0008), xylulose (AUC, 0.731; *p* = 0.001), ribulose (AUC, 0.727; *p* = 0.001), N-acetyl-D-glucosamine (AUC, 0.722; *p* = 0.006), and indoleacrylic acid (AUC, 0.692; *p* = 0.01), were significantly associated with NC controls (Figure 4E). Therefore, these results suggest the potential diagnostic value of the fecal metabolic signature in CRC risk stratification. These results thus illustrate the changing energy metabolism by gut microbes in CRC patients.

### Correlation Between the Altered Metabolites and Gut Microbiome

**FIGURE 5:**
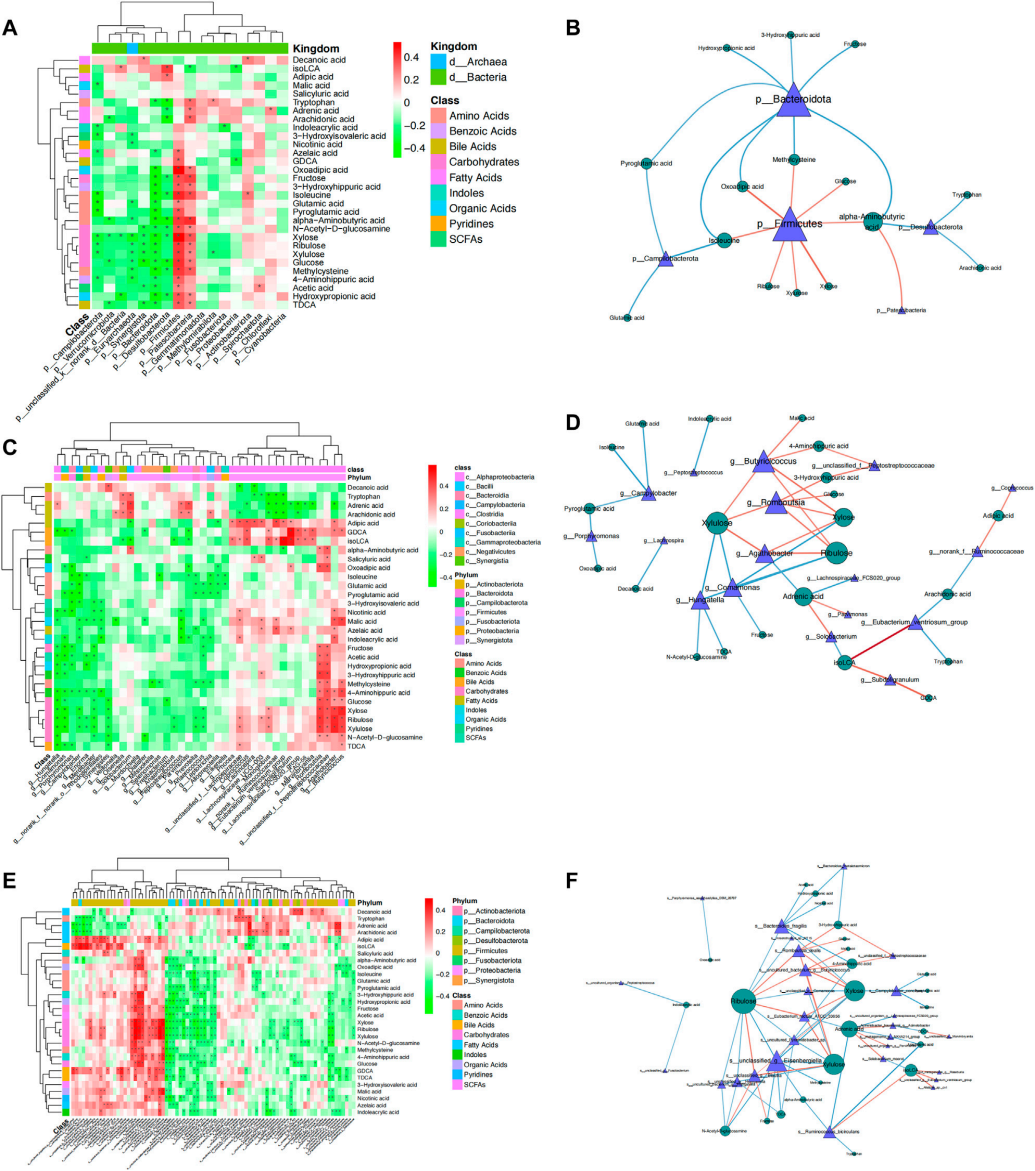
Spearman’s rank correlation between metabolites and gut microbiota in CRC and NC groups. A network diagram and heatmap demonstrating the correlation between differential metabolites and bacteria at the phylum, genus, and species levels. Spearman rank correlation coefficient, *: *p* < 0.05. Red represents a positive correlation, while blue and green represent a negative correlation.

To further investigate the correlation between gut microbial dysbiosis and altered fecal metabolites, Spearman’s correlation coefficients were calculated between the 29 potential metabolic biomarkers and microbial communities at the phylum, genus, and species levels. At the phylum level, Firmicutes was positively correlated with the fermentation of carbohydrates and amino acids, and Bacteroidetes was negatively correlated with the use of carbohydrates, amino acids, and organic acids. *Campilobacterota*, which causes bacterial diarrheal illness, was negatively correlated with pyroglutamic acid and glutamic acid, while *Desulfobacterota* was negatively correlated with tryptophan, arachidonic acid, and alpha-aminobutyric acid, and *Patescibacteria* was positively correlated with alpha-aminobutyric acid (Figures 5A, B). At the genus level, 29 bacterial taxa were significantly associated with 22 fecal metabolites (Figures 5C, D). At the species level, we further explored the significantly differentially abundant bacterial-associated metabolic biomarkers. The results revealed that *Parvimonas* was positively correlated with the production of adrenic acid, and *Fusobacterium* was negatively correlated with indoleacrylic acid, which are both CRC-associated oral pathogens that have been known for a long time (Sun J et al., 2020). *Ruminococcus bicirculans*, a new Firmicutes species belonging to the dominant human colonic microbiota (Wegmann et al., 2014), was negatively correlated with arachidonic acid and tryptophan. In addition, we focused on *Bacteroides fragilis* because it promotes CRC progression via multiple mechanisms, such as weakening adherence junctions, breakdown of the extracellular matrix, and reorganization of the cytoskeleton (Li et al., 2021). Enterotoxigenic *Bacteroides fragilis* (ETBF) produces *Bacteroides fragilis* toxin, which has been associated with acute diarrheal disease, inflammatory bowel disease, and colorectal cancer (CRC) (Henstra et al., 2021). In this study, we found that *Bacteroides fragilis* was significantly enriched in the CRC group, showing moderate to strong negative correlations with the metabolism of ribulose, xylose, hydroxypropionic acid, 4-aminohippuric acid, nicotinic acid, and acetic acid. In contrast, *Blautia* was significantly increased in the NC group and showed moderate positive correlations with ribulose, N-acetyl-D-glucosamine xylulose, and fructose (Figures 5E, F). Collectively, these results showed a strong correlation between gut microbiome disorders and metabolic alterations in CRC patients, suggesting that CRC may cause gut microbiota dysbiosis, which results in dramatic shifts in host metabolite abundance.

## Discussion

The gut microbiome is essential for health, while dysbiosis is related to various diseases, including metabolic syndrome, diabetes, obesity, neurodegenerative disease, and cancers, including colorectal carcinogenesis (Sheikh et al., 2021). A healthy gut environment is characterized by a balance of bacterial communities and their favorable metabolic action (Nie et al., 2019). The effects of gut microbiota on health and disease modulation are mainly through their metabolites, such as secondary bile salts from primary bile salts, hydrogen sulfide, TMAO, and indoxyl sulfate, which may promote chronic inflammation and facilitate CRC (Han et al., 2020). Specifically, the intricate interplay among the intestinal flora, metabolites, chronic inflammation or immune responses, host genetics, and essential environmental factors jointly contributes to the development of colon tumors (Chen and Chen, 2021). Bacterial metabolites may serve as nutrients harvested from debris in the colon and may have protective roles in maintaining the epithelial barrier and immune homeostasis. For instance, SCFAs are well-known for their potential to stimulate regulatory T cells to maintain the intestinal tolerogenic state (Kim, 2021). In contrast, an unbalanced diet and host intestinal transformation may produce detrimental gut metabolites to promote cancer progression (Shock et al., 2021).

In this study, through a combination of 16S rDNA sequencing and UPLC–MS/MS techniques, we investigated the correlation between the microbiota and their metabolites in CRC patients and NC controls. Through these approaches, we found that both the diversity and richness of gut flora were reduced in CRC patients, and we also identified specific bacteria in association with CRC. In line with a previous report (Gagnière et al., 2016), we also detected higher levels of *Campylobacter*, Bacteroidetes, and Fusobacteria in CRC patients with local recruitment, suggesting that common core microbes are associated with CRC development. Moreover, the *Prevotella* genus and species of *Bacteroides fragilis* are known for their roles in promoting inflammation and CRC development (Bundgaard-Nielsen et al., 2019), which was further confirmed in this study. We noticed that decreased levels of monosaccharides are related to the production of long-chain fatty acids in association with decreased levels of Firmicutes in CRC patients. We, therefore, speculate that the alteration in mucosal carbohydrate availability may be caused by the disruption of the resident microbiota, which could be a possible mechanism employed by the microbiota for tumor growth (Rakoff-Nahoum et al., 2014). Conversely, *Blautia* and *Lachnospiracaea* were significantly enriched in NC controls. *Blautia* is a genus of anaerobic bacteria with probiotic characteristics, which may be beneficial for host health and alleviate metabolic syndrome. Conversely, *Fusobacterium*, *Bacteroides*, *Parvimonas,* and *Prevotella* along with increased Firmicutes were associated with CRC and hepatocellular carcinoma patients, while the underlying mechanism remains elusive (Chen T et al., 2021; Liu X et al., 2021). Lachnospiraceae is the main producer of butyrate, which is known for its capacity to suppress the motility of colorectal cancer cells by deactivating Akt/ERK signaling in a histone deacetylase dependent manner (Li et al., 2017; Zhang et al., 2019). In our study, the abundance of *Blautia* and Lachnospiraceae was lower in CRC fecal samples, which may reflect the unique environmental and genetic background of our local recruitment.

Cancer cells have an altered metabolic state that supports their growth, for example, aerobic glycolysis, known as the Warburg effect. CRC cells were reported to exhibit the Warburg effect leading to dysfunctional mitochondria (Ohshima et al., 2022). In our study, the levels of many monosaccharides, such as xylose, xylulose, ribulose, and N-cetyl-D-glucosamine, were relatively low in CRC patients, which may reflect the enhanced glycolysis and production of fatty acids, such as adipic acid, adrenic acid, arachidonic acid, and azelaic acid. Similar results were found in mouse liver cancer models, and one study showed that a switch from oxidative phosphorylation to glycolysis in the Warburg effect was invariably accompanied by a marked decline in fatty acid oxidation and a reciprocal increase in the activity of pyruvate dehydrogenase, which links glycolysis to the TCA cycle (Wang et al., 2019). This study also found that the survival benefits of high-fat diets in cancer patients are due to a reversal of the Warburg effect and other tumor-associated metabolic and cell cycle abnormalities. Thus, it is possible that fatty acid oxidation may inhibit glycolysis in cancer cells. Tryptophan catabolism in cancer is recognized as an important microenvironment modulator that alleviates antitumor immune responses. It has been reported that cancer cells create an immunosuppressive milieu in tumors and tumor-draining lymph nodes by inducing T cell anergy and apoptosis through depletion of tryptophan and accumulation of immunosuppressive tryptophan catabolites (Fiore and Murray, 2021). In our study, fecal tryptophan was significantly increased in CRC patients. It has been reported that the fecal tryptophan indole pathway is impaired in colorectal cancer and that indole-related bacteria may lead to downregulation of the tryptophan indole metabolic pathway (Sun XZ et al., 2020). We noticed a linkage of increased levels of fecal tryptophan associated with *Ruminococcus bicirculans* and *Fusobacterium* in our study for decreased production of indole*.* Arachidonic acid is an important polyunsaturated fatty acid for cell signaling, and cells metabolize arachidonic acid to adrenic acid *via* 2-carbon elongation reactions. Similar to arachidonic acid, adrenic acid can be converted into multiple oxygenated metabolites, which are involved in various physiological and pathophysiological processes (Galano et al., 2015), such as iron-catalyzed necrosis, referred to as ferroptosis (Lee et al., 2020). Iron-dependent cancer cells are more vulnerable to ferroptosis (Hassannia et al., 2019). In this study, we observed that arachidonic acid and adrenic acid were strongly upregulated in CRC patients and were positively correlated with the pathogenic bacteria (e.g., *Parvimonas*, *Solobacterium moorei*, and *Acinetobacter baumannii*). These data may illustrate that the metabolite changes that are modulated by the gut microbiota could be involved in ferroptosis. Decanoic acid, an upregulated metabolite, is a valuable diagnostic biomarker for early CRC (Crotti et al., 2016; Uchiyama et al., 2017), which is in concordance with our data.

Although we detected a disease-associated signature in CRC fecal specimens, these findings must be viewed as preliminary and limited, and the difference in metabolite abundance needs to be validated in a larger independent cohort. Another limitation is that we did not collect detailed information on dietary intake, which may be an additional confounder to our data. Despite these limitations, we were able to describe the alterations of the gut microbiome and fecal metabolome in CRC patients in comparison with healthy volunteers, and further documentation may have the potential for the prevention, diagnosis, and treatment of CRC.

## Conclusion

We present evidence showing altered metabolites and the microbiome in CRC feces, and we also demonstrated the correlation between gut microbial dysbiosis and altered fecal metabolites. Specifically, we identified several bacterial species (e.g., *Bacteroides fragilis* and *Veillonella parvula*) strongly associated with colorectal cancer development and hypothesized that fatty acid oxidation may inhibit glycolysis in cancer cells. Meanwhile, we also noticed that the metabolite changes that are modulated by the gut microbiota could be involved in ferroptosis and a linkage of increased levels of fecal tryptophan with *Ruminococcus bicirculans* and *Fusobacterium*. In conclusion, our results support the notion that fecal microbial and metabolic biomarkers have great potential for clinical practice and that further studies are needed to investigate the role of such biomarkers in CRC diagnostics and management.

## Data Availability

The 16S datasets presented in this study can be found in online repositories. The names of the repository/repositories and accession number(s) can be found below: NCB SRA(https://www.ncbi.nlm.nih.gov/), which can be accessed with the BioProject identifier PRJNA824020.
