# Supplementary material for: Alterations of the Gut Microbiome and Fecal Metabolome in Colorectal Cancer: Implication of Intestinal Metabolism for Tumorigenesis
Source: Front Physiol. 2022 May 4;13:854545. doi: 10.3389/fphys.2022.854545 (PMC9116530; doi:10.3389/fphys.2022.854545)
Supplement: Supplementary file 1 [file DataSheet1.docx]

Supplementary Material

Figure S1


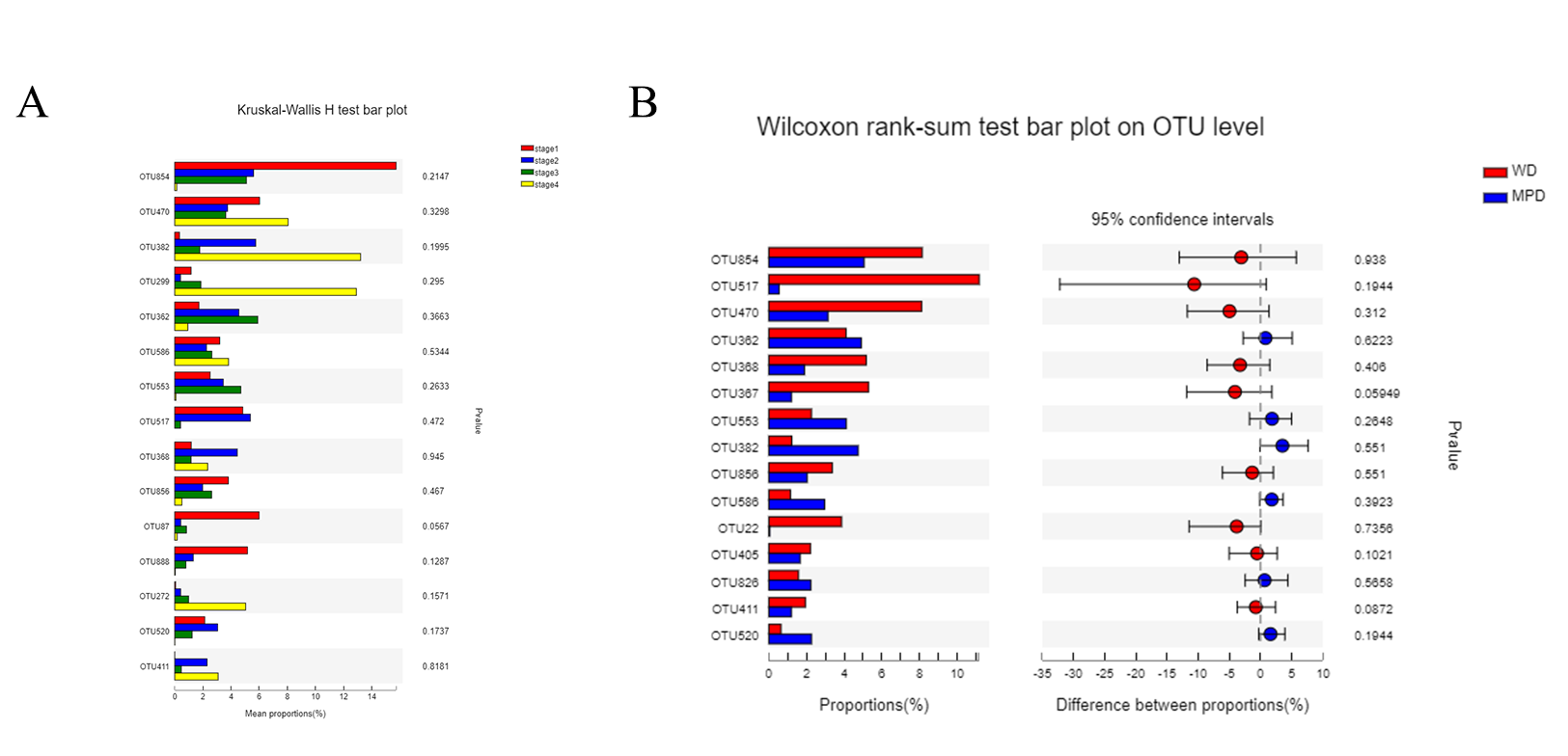
**Supplementary Figure 1.** Relative abundance of the bacterial composition in CRC at different differentiation status and pTNM stage.

Figure S2


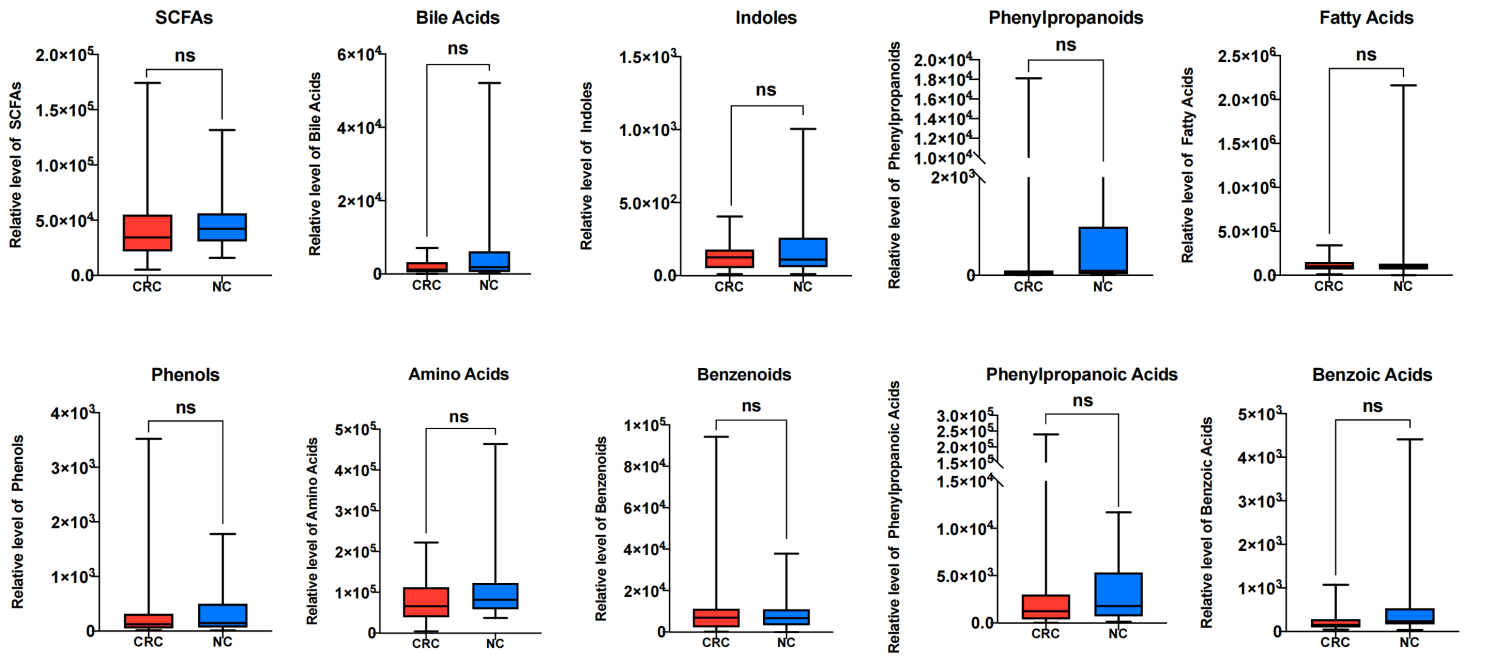


**Supplementary Figure 2.** Quantification of part fecal metabolite in colorectal cancer patients and normal colorectal mucous membrane controls.

Figure S3


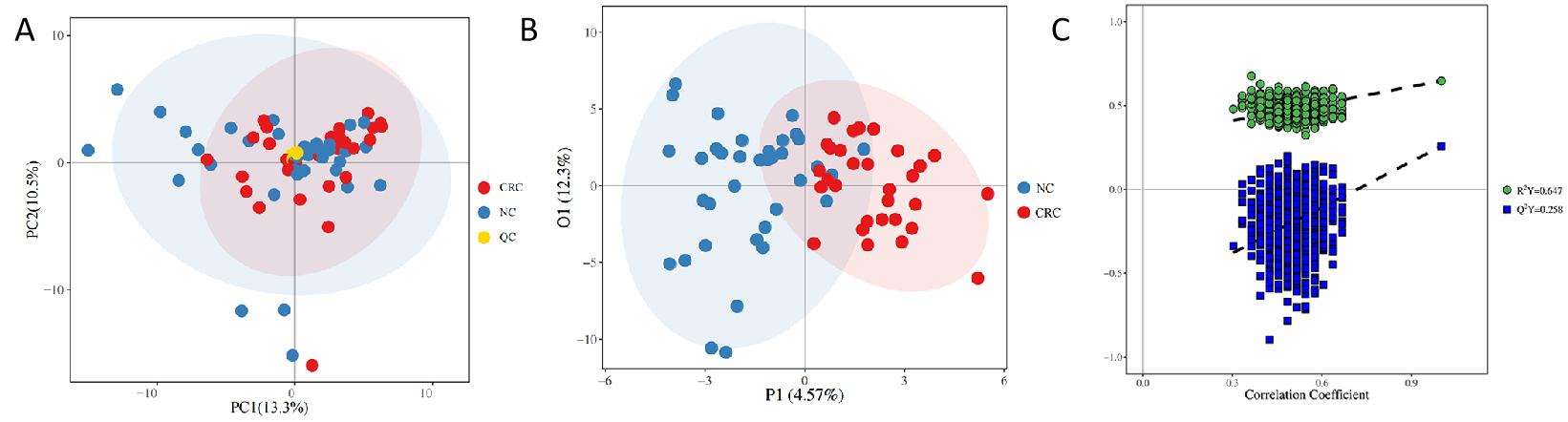


**Supplementary Figure 3. (A)** PCA scores plot of fecal specimens based on NC group (blue dots) and CRC group (red dots). **(B)** Score plot in the OPLS-DA model for CRC group (red dots) and NC group (blue dots). **(C)** Statistical validation of the corresponding OPLS-DA model by permutation analysis (200 times). R^2^ is the explained variance, and Q^2^ is the predictive ability of the model. PCA, Principal Component Analysis; OPLS-DA, orthogonal partial least squares-discriminant analysis; CRC, colorectal cancer; NC, normal colorectal mucous membrane; QC, quality control.

Figure S4


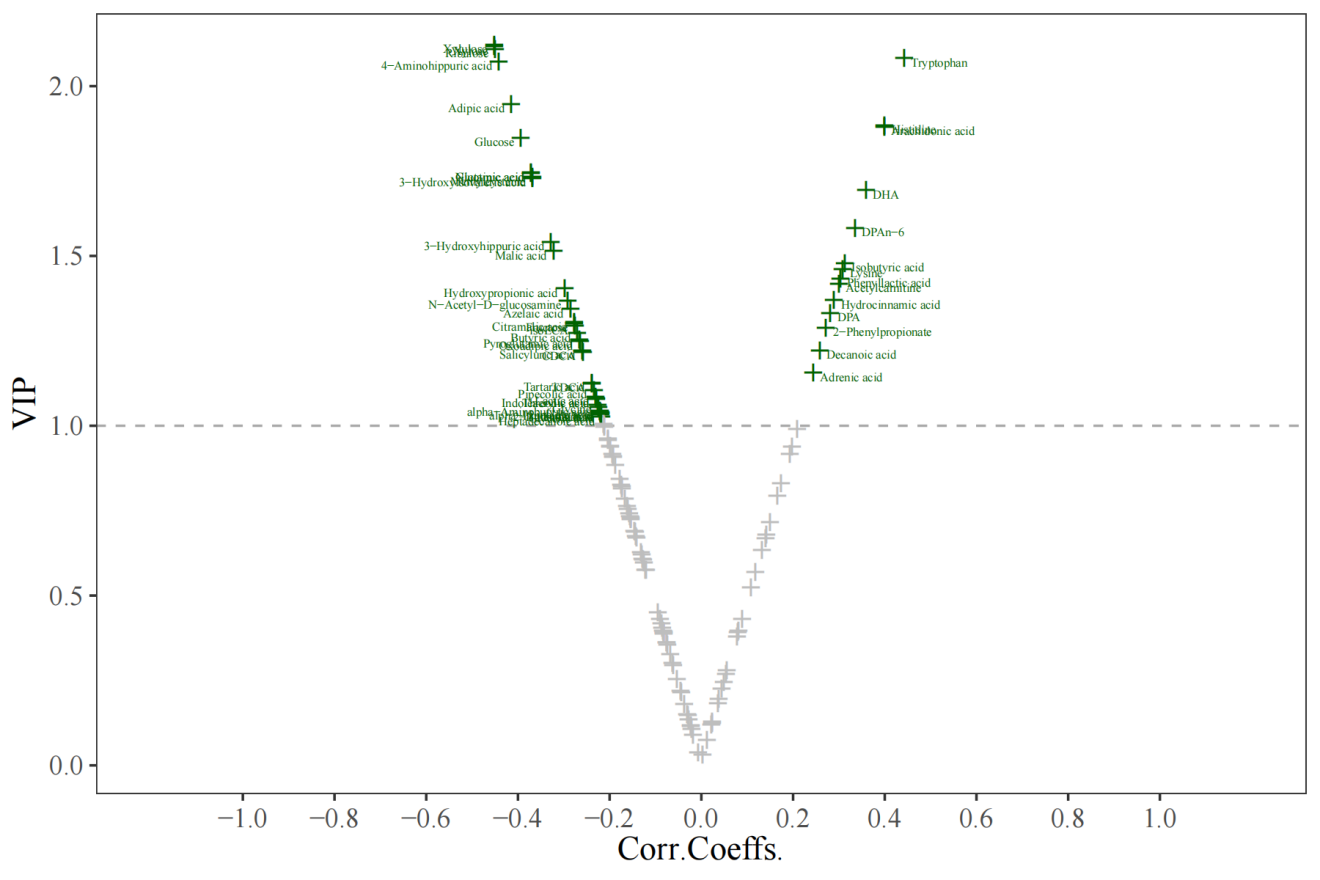


**Supplementary Figure 4.** Volcano plot for analyses of metabolites from colorectal cancer group and normal colorectal mucous membrane group. variable importance of the projection scores > 1 and P < 0.05 are marked in green, and the rest are in grey.

Figure S5


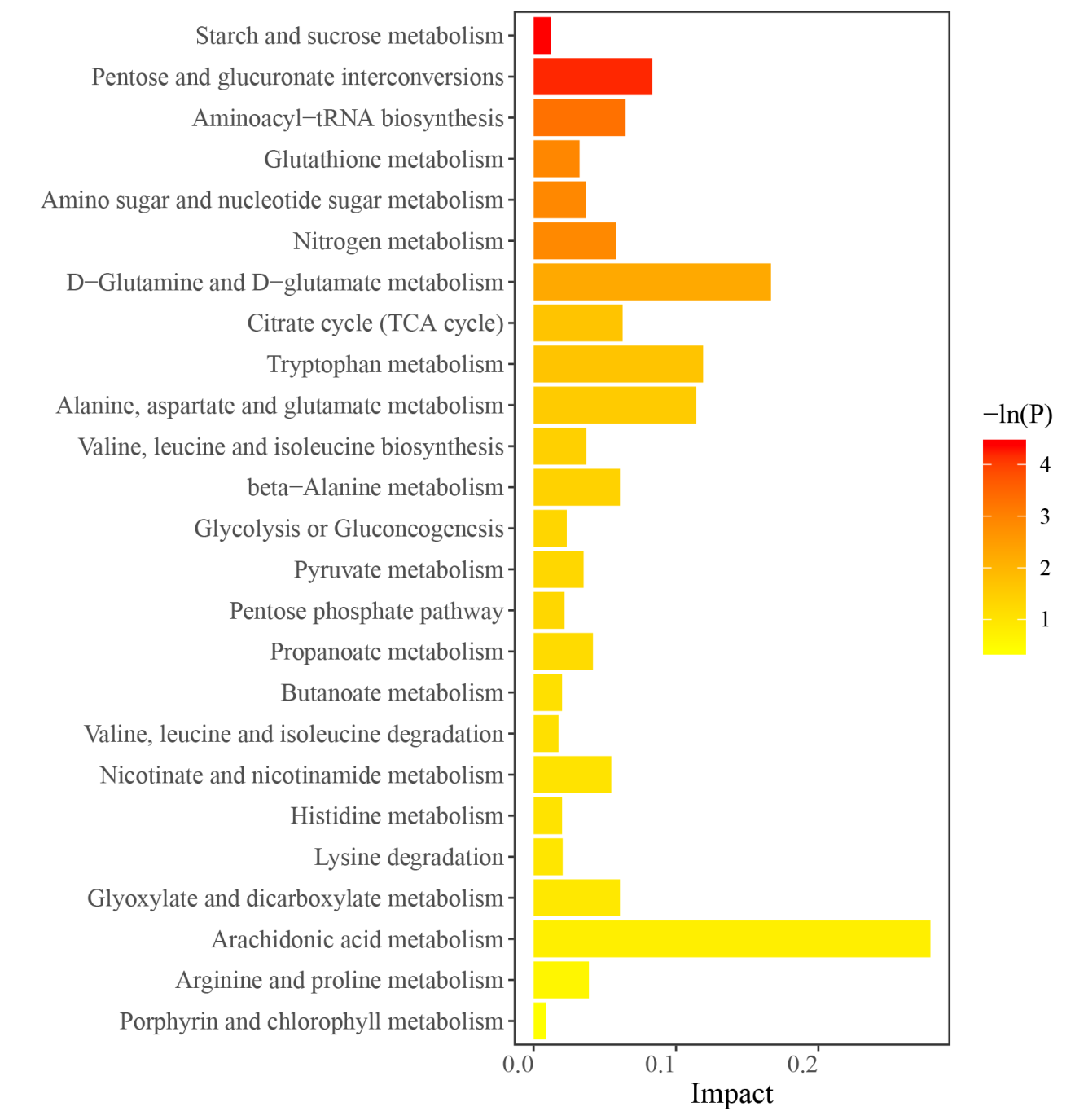


**Supplementary Figure 5.** Metabolic pathway analysis in differential metabolites.

**Table S1. The number of specific sequences for each sample**

| Sample\Info | Seq_num | | Base_num | | | Mean_length | Min_length | | | Max_length | | |
| --- | --- | --- | --- | --- | --- | --- | --- | --- | --- | --- | --- | --- |
| A1 | 56798 | | 14539666 | | | 255.9890489 | 253 | | | 315 | | |
| A2 | 47587 | | 12191310 | | | 256.1899258 | | 250 | | 259 | | |
| A3 | 51857 | | 13271741 | | | 255.9295948 | | 247 | | 257 | | |
| A4 | 56737 | | 14519645 | | | 255.9113982 | | 241 | | 315 | | |
| A5 | 54146 | | 13855994 | | | 255.9006021 | | 248 | | 259 | | |
| A6 | 54124 | | 13847133 | | | 255.8409024 | | 212 | | 258 | | |
| A7 | 60524 | | 15490950 | | | 255.9472275 | | 250 | | 259 | | |
| A8 | 57995 | | 14844192 | | | 255.95641 | | 212 | | 259 | | |
| A9 | 60045 | | 15360147 | | | 255.8105921 | | 212 | | 259 | | |
| A10 | 51727 | | | 13239724 | 255.9538346 | | | | 252 | | 284 |  |
| A11 | 56107 | | | 14358487 | 255.9125778 | | | | 250 | | 258 |  |
| A12 | 53419 | | | 13641483 | 255.367622 | | | | 206 | | 259 |  |
| A13 | 61597 | | | 15763974 | 255.9211325 | | | | 249 | | 259 |  |
| A14 | 52994 | | | 13564957 | 255.9715628 | | | | 248 | | 258 |  |
| A15 | 56682 | | | 14505433 | 255.9089835 | | | | 212 | | 268 |  |
| A16 | 55599 | | | 14214350 | 255.6583752 | | | | 251 | | 300 |  |
| A17 | 60247 | | | 15415170 | 255.8661842 | | | | 244 | | 267 |  |
| A18 | 57348 | | | 14670527 | 255.8158436 | | | | 212 | | 269 |  |
| A19 | 59842 | | | 15312496 | 255.8820895 | | | | 250 | | 259 |  |
| A20 | 48602 | | | 12436814 | 255.8909921 | | | | 243 | | 258 |  |
| A21 | 52822 | | | 13509034 | 255.7463557 | | | | 249 | | 341 |  |
| A22 | 51390 | | | 13153471 | 255.9539015 | | | | 216 | | 258 |  |
| A23 | 60707 | | | 15533545 | 255.8773288 | | | | 231 | | 258 |  |
| A24 | 46924 | | | 12007447 | 255.8913775 | | | | 251 | | 258 |  |
| A25 | 73412 | | | 18786754 | 255.9084891 | | | | 216 | | 315 |  |
| A26 | 64307 | | | 16441609 | 255.6737058 | | | | 212 | | 284 |  |
| A27 | 56840 | | | 14546292 | 255.9164673 | | | | 212 | | 259 |  |
| A28 | 74489 | | | 19057567 | 255.8440441 | | | | 206 | | 329 |  |
| A29 | 70503 | | | 18041857 | 255.9019758 | | | | 206 | | 321 |  |
| A30 | 69709 | | | 17824064 | 255.6924357 | | | | 204 | | 289 |  |
| B1 | 64967 | | | 16624287 | 255.888174 | | | | 250 | | 261 |  |
| B2 | 57927 | | | 14825909 | 255.9412536 | | | 221 | | 262 | |  |
| B3 | 54217 | | | 13876887 | 255.9508457 | | | 221 | | 259 | |  |
| B4 | 54654 | | | 13985588 | 255.8932192 | | | 212 | | 315 | |  |
| B5 | 59622 | | | 15253926 | 255.8439167 | | | 200 | | 259 | |  |
| B6 | 54828 | | | 14036131 | 256.0029729 | | | 251 | | 296 | |  |
| B7 | 64929 | | | 16611117 | 255.8350968 | | | 212 | | 315 | |  |
| B8 | 56255 | | | 14397567 | 255.933997 | | | 204 | | 259 | |  |
| B9 | 56087 | | | 14352356 | 255.894521 | | | 216 | | 285 | |  |
| B10 | | 57246 | | 14645989 | | 255.8430109 | 212 | | | 259 | |  |
| B11 | | 47659 | | 12185612 | | 255.6833337 | 250 | | | 259 | |  |
| B12 | | 57735 | | 14774777 | | 255.9067637 | 212 | | | 259 | |  |
| B13 | | 54856 | | 14036097 | | 255.8716822 | 249 | | | 259 | |  |
| B14 | | 47210 | | 12083782 | | 255.9581021 | 249 | | | 259 | | |
| B15 | | 61983 | | 15854688 | | 255.7909104 | 236 | | | 258 | | |
| B16 | | 53989 | | 13817578 | | 255.9332086 | 250 | | | 258 | | |
| B17 | | 62888 | | 16097776 | | 255.9753212 | 250 | | | 259 | | |
| B18 | | 52317 | | 13384452 | | 255.8337061 | 251 | | | 259 | | |
| B19 | | 57890 | | 14801010 | | 255.6747279 | 211 | | | 258 | | |
| B20 | | 49222 | | 12598658 | | 255.9558328 | 236 | | | 258 | | |
| B21 | | 59880 | | 15322864 | | 255.8928524 | 212 | | | 258 | | |
| B22 | | 62069 | | 15881686 | | 255.8714656 | 223 | | | 259 | | |
| B23 | | 64243 | | 16443812 | | 255.9627041 | 244 | | | 315 | | |
| B24 | | 54838 | | 14016982 | | 255.6070973 | 206 | | | 267 | | |
| B25 | | 60479 | | 15478716 | | 255.9353825 | 250 | | | 335 | | |
| B26 | | 54812 | | 14026990 | | 255.9109319 | 212 | | | 260 | | |
| B27 | | 59281 | | 15170226 | | 255.9036791 | 230 | | | 259 | | |
| B28 | | 64996 | | 16633372 | | 255.9137793 | 207 | | | 258 | | |
| B29 | | 50604 | | 12946737 | | 255.8441428 | 212 | | | 306 | | |
| B30 | | 55620 | | 14233939 | | 255.9140417 | 243 | | | 341 | | |
| B31 | | 51484 | | 13156837 | | 255.5519579 | 212 | | | 308 | | |
| B32 | | 60390 | | 15449434 | | 255.8276867 | 217 | | | 268 | | |
| B33 | | 67223 | | 17200135 | | 255.8668164 | 219 | | | 262 | | |
| B34 | | 67926 | | 17380118 | | 255.8684156 | 206 | | | 302 | | |
| B35 | | 67407 | | 17250665 | | 255.9180055 | 207 | | | 302 | | |
| B36 | | 53095 | | 13572700 | | 255.6304737 | 204 | | | 268 | | |

A, colorectal cancer group; B, human with normal colorectal mucous membrane group

**Table S2. Differences of α‐diversity between CRC and NC groups**

| Estimators | CRC-Mean | CRC-Sd | NC-Mean | NC-Sd | *P-*value |
| --- | --- | --- | --- | --- | --- |
| sobs | 237.83 | 90.867 | 245.33 | 67.202 | 0.7015 |
| shannon | 3.087 | 0.62652 | 3.3709 | 0.51115 | 0.04675 |
| simpson | 0.11098 | 0.078564 | 0.080224 | 0.043721 | 0.04894 |
| ace | 283.84 | 102.49 | 292.28 | 76.814 | 0.7035 |
| chao | 285.52 | 106.13 | 292.63 | 83.646 | 0.7618 |
| coverage | 0.99905 | 0.00035261 | 0.99902 | 0.00031052 | 0.6692 |

CRC, colorectal cancer; NC, human with normal colorectal mucous membrane

**Table S3. Metabolic pathway analysis in differential metabolites.**

|  | Total In Pathway | Expected | Hits | Raw *P* | ^'^-ln(*p*) | Holm *P* | FDR | Impact | Enriched Compounds |
| --- | --- | --- | --- | --- | --- | --- | --- | --- | --- |
| Starch and sucrose metabolism | 50 | 0.49855 | 3 | 0.012559 | 4.3773 | 1 | 0.58877 | 0.0122 | Fructose Glucose Xylose |
| Pentose and glucuronate interconversions | 53 | 0.52846 | 3 | 0.014719 | 4.2186 | 1 | 0.58877 | 0.08333 | Ribulose Xylulose Xylose |
| Aminoacyl-tRNA biosynthesis | 75 | 0.74782 | 3 | 0.036762 | 3.3033 | 1 | 0.75245 | 0.06452 | Isoleucine Tryptophan Glutamic acid |
| Glutathione metabolism | 38 | 0.37889 | 2 | 0.053862 | 2.9213 | 1 | 0.75245 | 0.03226 | Glutamic acid Pyroglutamic acid |
| Amino sugar and nucleotide sugar metabolism | 88 | 0.87744 | 3 | 0.054905 | 2.9021 | 1 | 0.75245 | 0.03669 | N-Acetyl-D-glucosamine Fructose Xylose |
| Nitrogen metabolism | 39 | 0.38887 | 2 | 0.056434 | 2.8747 | 1 | 0.75245 | 0.05769 | Tryptophan Glutamic acid |
| D-Glutamine and D-glutamate metabolism | 11 | 0.10968 | 1 | 0.10458 | 2.2578 | 1 | 1 | 0.16667 | Glutamic acid |
| Citrate cycle (TCA cycle) | 20 | 0.19942 | 1 | 0.18227 | 1.7023 | 1 | 1 | 0.0625 | Malic acid |
| Tryptophan metabolism | 79 | 0.7877 | 2 | 0.18502 | 1.6873 | 1 | 1 | 0.11904 | Tryptophan Oxoadipic acid |
| Alanine, aspartate and glutamate metabolism | 24 | 0.2393 | 1 | 0.21468 | 1.5386 | 1 | 1 | 0.11429 | Glutamic acid |
| Phenylalanine, tyrosine and tryptophan biosynthesis | 27 | 0.26921 | 1 | 0.23818 | 1.4347 | 1 | 1 | 0 | Tryptophan |
| Valine, leucine and isoleucine biosynthesis | 27 | 0.26921 | 1 | 0.23818 | 1.4347 | 1 | 1 | 0.03704 | Isoleucine |
| beta-Alanine metabolism | 28 | 0.27919 | 1 | 0.24586 | 1.403 | 1 | 1 | 0.06061 | Hydroxypropionic acid |
| Glycolysis or Gluconeogenesis | 31 | 0.3091 | 1 | 0.26847 | 1.315 | 1 | 1 | 0.02326 | Glucose |
| Lysine biosynthesis | 32 | 0.31907 | 1 | 0.27586 | 1.2879 | 1 | 1 | 0 | Oxoadipic acid |
| Pentose phosphate pathway | 32 | 0.31907 | 1 | 0.27586 | 1.2879 | 1 | 1 | 0.02174 | Glucose |
| Pyruvate metabolism | 32 | 0.31907 | 1 | 0.27586 | 1.2879 | 1 | 1 | 0.03509 | Malic acid |
| Propanoate metabolism | 35 | 0.34898 | 1 | 0.2976 | 1.212 | 1 | 1 | 0.04167 | Hydroxypropionic acid |
| Valine, leucine and isoleucine degradation | 40 | 0.39884 | 1 | 0.33245 | 1.1013 | 1 | 1 | 0.01754 | Isoleucine |
| Butanoate metabolism | 40 | 0.39884 | 1 | 0.33245 | 1.1013 | 1 | 1 | 0.02 | Glutamic acid |
| Histidine metabolism | 44 | 0.43872 | 1 | 0.35913 | 1.0241 | 1 | 1 | 0.02 | Glutamic acid |
| Nicotinate and nicotinamide metabolism | 44 | 0.43872 | 1 | 0.35913 | 1.0241 | 1 | 1 | 0.05455 | Nicotinic acid |
| Lysine degradation | 47 | 0.46863 | 1 | 0.37847 | 0.97162 | 1 | 1 | 0.02041 | Oxoadipic acid |
| Glycine, serine and threonine metabolism | 48 | 0.4786 | 1 | 0.38479 | 0.95506 | 1 | 1 | 0 | Tryptophan |
| Fatty acid biosynthesis | 49 | 0.48857 | 1 | 0.39105 | 0.93893 | 1 | 1 | 0 | Decanoic acid |
| Glyoxylate and dicarboxylate metabolism | 50 | 0.49855 | 1 | 0.39725 | 0.9232 | 1 | 1 | 0.06061 | Malic acid |
| Arachidonic acid metabolism | 62 | 0.6182 | 1 | 0.46706 | 0.76129 | 1 | 1 | 0.27869 | Arachidonic acid |
| Arginine and proline metabolism | 77 | 0.76776 | 1 | 0.54348 | 0.60976 | 1 | 1 | 0.03883 | Glutamic acid |
| Porphyrin and chlorophyll metabolism | 104 | 1.037 | 1 | 0.65536 | 0.42257 | 1 | 1 | 0.00877 | Glutamic acid |

**Table S4.The differential metabolites in CRC at different pTNM stage**

| **Metabolite** | **Class** | **HMDB** | **KEGG** | ***P*-value** | **FDR** |
| --- | --- | --- | --- | --- | --- |
| 4-Hydroxyphenylpyruvic acid | Phenols | HMDB0000707 | C01179 | 0.0128 | 0.5991 |
| Butyric acid | SCFAs | HMDB0000039 | C00246 | 0.007 | 0.5991 |
| Carnitine | Carnitines | HMDB0000062 | C00318 | 0.0286 | 0.5991 |
| Acetic acid | SCFAs | HMDB0000042 | C00033 | 0.0283 | 0.5991 |
| Ethylmethylacetic acid | SCFAs | HMDB0002176 | C18319 | 0.0388 | 0.5991 |
| Indole-3-carboxylic acid | Indoles | HMDB0003320 | C19837 | 0.0139 | 0.5991 |
| Isovaleric acid | SCFAs | HMDB0000718 | C08262 | 0.0375 | 0.5991 |
| Phenylpyruvic acid | Benzenoids | HMDB0000205 | C00166 | 0.0208 | 0.5991 |
